# The Alzheimer’s Disease Sequencing Project – Discovery, Discovery Extension and Follow Up Study (ADSP‐FUS): *APOE* genotype status and demographic characteristics across datasets

**DOI:** 10.1002/alz.092629

**Published:** 2025-01-03

**Authors:** Pedro R. Mena, Andrew F Zaman, Kelley M. Faber, Larry D. Adams, Jovita D. Inciute, Patrice L. Whitehead, Tatiana M. Foroud, Dolly Reyes‐Dumeyer, Amanda B Kuzma, Heather Issen Nicaretta, Adam C. Naj, Eden R. Martin, Clifton L. Dalgard, Gerard D. Schellenberg, Li‐San Wang, Richard Mayeux, Badri N. Vardarajan, Jeffery M. Vance, Michael L. Cuccaro, Brain Kunkle, Margaret A Pericak‐Vance

**Affiliations:** ^1^ John P. Hussman Institute for Human Genomics, University of Miami Miller School of Medicine, Miami, FL USA; ^2^ National Centralized Repository for Alzheimer’s Disease and Related Dementias (NCRAD), Indianapolis, IN USA; ^3^ John P. Hussman Institute for Human Genomics, Miami, FL USA; ^4^ John P. Hussman Institute for Human Genomics, University of Miami Miller School of Medicine, Miami, FL, USA, Miami, FL USA; ^5^ The Taub Institute for Research on Alzheimer’s Disease and the Aging Brain, Vagelos College of Physicians & Surgeons, Columbia University, New York, NY USA; ^6^ University of Pennsylvania Perelman School of Medicine, Philadelphia, PA USA; ^7^ Perelman School of Medicine, University of Pennsylvania, Philadelphia, PA USA; ^8^ Department of Anatomy, Physiology, and Genetics, Uniformed Services University of the Health Sciences, Bethesda, MD USA; ^9^ The Taub Institute for Research on Alzheimer’s Disease and The Aging Brain, Columbia University Medical Center and The New York Presbyterian Hospital, New York, NY USA; ^10^ The Taub Institute for Research on Alzheimer’s Disease and The Aging Brain, Columbia University, New York, NY USA; ^11^ 1501 NW 10th Avenue, Miami, FL USA

## Abstract

**Background:**

The ADSP is a National Institute on Aging (NIA) initiative focused on identifying genetic risk and protective variants for Alzheimer Disease (AD). Initial phases (Discovery and Discovery Extension) were predominantly non‐Hispanic Whites of European Ancestry (NHW‐EA). The ADSP expanded the population diversity in the Follow Up Study (ADSP‐FUS), and the current phase, ADSP‐FUS 2.0: The Diverse Population Initiative, focusing on whole genome sequencing (WGS) of non‐European populations including Hispanic/Latino (HL), non‐Hispanic Black with African Ancestry (NHB‐AA) and Asian populations. Support for these efforts include newly funded initiatives such as The DAWN Project, focused on recruitment of African, African‐American and Hispanic American populations, and the Asian Cohort for Alzheimer’s Disease (ACAD).

**Methods:**

ADSP cohorts consist of studies of AD, dementia, and age‐related conditions. Clinical classifications are assigned based on standard criteria and derived from clinical measures and history, as well as additional neuropathologic data. In addition to production of WGS, *APOE* genotyping is available for all ADSP samples.

**Results:**

The ADSP currently consists of 40 cohorts comprised of ∼36,300 individuals, with plans to sequence >110,000 individuals from diverse race/ethnicity. Genotyping, sequencing, and clinical adjudication has been performed on 36,361 participants (cases N = 12,133, median age = 72; cognitively‐unimpaired(CU) individuals N = 17,116, median age = 74; ADRD N = 7,112, median age = 71). Mean ages for cases and controls vary across cohorts, 57.0+5.6 to 86.5+4.2 cases and 63.3+7.8 to 90.0+0 controls. 61% participants female, distributed as follows: cases(60.3%), CU(63.7%), and ADRD(55.8%). *APOE* genotype proportions differ considerably across reported race/ethnicity, for example highest for *APOE ε*4/*ε*4 carriers observed in Non‐Hispanic whites participants (7.4%) and the lowest in Asians (1.7%)

**Conclusion:**

The results provide an overview of clinical features in ADSP cohorts. The growth of the ADSP‐FUS 2.0 is central to the ADSP and expanding the size and diversity of this genomic resource available via NIAGADS. WGS data will be integrated with ADSP programs focused on phenotype harmonization, association analyses, functional genomics, and machine learning. In concert with these programs, the ADSP‐FUS 2.0 will accelerate the identification and understanding of potential genetic risk and protective variants for AD across all populations with the target of developing new treatments that are globally effective.

